# Longitudinal Associations Between Autistic Traits, Self-compassion, Anxiety and Depression in Autistic and Non-autistic Adults Without Intellectual Disability

**DOI:** 10.1007/s10803-023-06157-6

**Published:** 2023-10-24

**Authors:** John Galvin, Abby Howes, Gareth Richards

**Affiliations:** 1 Department of Psychology, University of Warwick, Coventry, UK; 2 Norwich Medical School, University of East Anglia, East Of England, UK; 3 School of Psychology, Newcastle University, Newcastle upon Tyne, UK

**Keywords:** Autism, Autistic Traits, Self-Compassion, Longitudinal, Anxiety, Depression

## Abstract

**Purpose:**

Previous cross-sectional research suggests self-compassion may mediate associations between autistic traits and mental health in autistic and non-autistic adults. However, no research to date has examined these relationships longitudinally. In this study, we used a cross-lagged panel analysis to examine correlations over time between autistic traits, self-compassion, and anxiety/depression.

**Methods:**

Participants were from the UK and included autistic (*n* = 228 at T1, *n* = 156 at T2, and *n* = 165 at T3) and non-autistic adults (*n* = 228 at T1, *n* = 122 at T2, and *n* = 124 at T3) without intellectual disability. Participants were recruited through an online participation platform and completed demographics, the Autism Spectrum Quotient (AQ), the Self-Compassion Scale (SCS), and the Hospital Anxiety and Depression Scale (HADS) at baseline (T1), 6 months (T2), and 12 months (T3).

**Results:**

In the autistic sample, negative correlations were observed between self-compassion and subsequent anxiety/depression across all models and timepoints, and these relationships were not reciprocal (i.e., earlier depression and anxiety did not predict future self-compassion). In the non-autistic sample, the findings generally suggested bi-directional relationships between self-compassion and anxiety/depression. In both groups, an indirect pathway between T1 autistic traits and T3 anxiety/depression via T2 self-compassion was confirmed.

**Conclusion:**

Considering the high prevalence of anxiety and depression among autistic people, and that self-compassion can be cultivated through practice, these findings suggest that self-compassion could be a useful therapeutic target to promote mental health in the autistic population.

Recent systematic reviews and meta-analyses have recorded increased prevalence of anxiety and depressive disorders in autistic populations (Hollocks et al., 2019; Lugo-Marin et al., 2019). Hollocks et al. (2019) reported the pooled estimation of current and lifetime prevalence for autistic adults ranged from 27 to 42% for anxiety disorder, and 23–37% for depressive disorder. Furthermore, the relationships between autistic traits (e.g., high attention to detail, difficulties with social skills, difficulties with communication, etc.) and anxiety and depression symptoms are well-documented (Baron-Cohen et al., 2001; Moss et al., 2015). At the time of writing, the identification of possible intervention targets that can improve the mental health of autistic people is the number one research priority of the autism community (Linden et al., 2023). Therefore, motivated by the link between self-compassion and mental health in non-autistic samples (Ferrari et al., 2019; Macbeth & Gumley, 2012), the current study investigated the longitudinal relationships between autistic traits, self-compassion, and anxiety/depression in a sample of autistic and non-autistic adults without intellectual disabilities.

According to researcher Neff (2003), self-compassion comprises a three-part process of self-kindness, common humanity, and mindfulness. *Self-kindness* involves treating oneself with the same kindness, concern, and support that we would offer a friend. It involves being understanding and gentle with oneself during times of suffering rather than self-critical or judgemental. *Common humanity* involves recognising that everyone confronts challenges and difficulties in life. Appreciating that suffering is a shared human experience can foster a sense of connectedness with others and alleviate feelings of isolation. *Mindfulness* involves being present and conscious of thoughts, feelings, and sensations without exaggeration, suppression, or avoidance. To be mindful involves observing and noticing negative thoughts and emotions with openness and clarity, not over-identifying with them, or being swept away by negative reactivity.

Several compassion-based therapies have been developed (e.g., Compassion-Focused Therapy [CFT]; Gilbert, 2009) that show evidence of moderate to strong positive changes in self-compassion, anxiety, and depression (Leaviss & Uttley, 2015; Wilson et al., 2019). According to Gilbert (2009), self-compassion activates a set of emotion regulation systems that promote feelings of contentment, safety, and connectedness. These feelings can help to regulate elevated threat-oriented emotions, such as shame and self-criticism, in individuals who are prone to these experiences. A surprising gap in the literature exists regarding autistic peoples’ experiences of self-compassion, including predictors and pathways to mental health disorders. Experiences such as bullying, trauma, and rejection from others are common among autistic individuals, as noted by various studies (Cage et al., 2018; Dodds, 2021; Humphrey & Hebron, 2015), and could contribute to a greater tendency towards self-blame and self-criticism in this already vulnerable group.

Recent attention has been given to the connection between autism/autistic traits and self-compassion (Cai & Brown, 2021; Cai et al., 2023a; Galvin et al., 2021; Galvin & Richards, 2023; Howes et al., 2021; Wilson et al., 2023). This research has revealed a negative relationship between autism/autistic traits and self-compassion, with autistic adults reporting lower levels of self-compassion compared to non-autistic adults (Cai et al., 2023a; Galvin & Richards, 2023). Lower self-compassion in autistic people is also associated with emotion regulation challenges (Cai et al., 2023b), and may be linked to exaggeration or suppression of emotional reactions (Garon et al., 2009), or the concealing or masking of autistic traits (Cook et al., 2021). In our recent cross-sectional study (Galvin & Richards, 2023), we reported an indirect pathway between autism/autistic traits and anxiety/depression symptoms via low levels of self-compassion in a sample of autistic and non-autistic adults. Notably, the strength of associations between autistic traits and anxiety/depression symptoms was significantly reduced (and no longer statistically significant in the autistic sample) once self-compassion was introduced as a mediating factor. Tentatively, we concluded that self-compassion may be a modifiable factor that can reduce the link between autistic traits and anxiety/depression.

## The Current Study

It should be noted that mental health difficulties in autistic people may result from and/or result in lower self-compassion. Longitudinal studies with general population samples have generally shown that self-compassion predicts subsequent depression and anxiety, but not the reverse (e.g., Pullmer et al., 2019; Stutts et al., 2018), suggesting low levels of self-compassion precede mental health symptoms rather than mental health symptoms precede self-compassion. However, the temporal sequencing of events has not yet been studied in autistic samples. The cross-sectional design of previous studies in this area (e.g., Cai et al., 2023a; Galvin & Richards, 2023) restricts the inferences that can be drawn. Should the current study reveal that self-compassion is associated with future anxiety and depression, it would offer initial indications that self-compassion holds promise as a pertinent focus for mental health intervention strategies in autistic adults. Additionally, determining a longitudinal mediation effect will help to characterise how autistic traits and self-compassion interact to influence future anxiety and depression.

The aim of the current study was therefore to test the interplay between autistic traits, self-compassion, and symptoms of anxiety and depression in a 12-month three-wave cross-lagged panel design. The study protocol at timepoint 1 (T1) was pre-registered on the Open Science Framework, and the findings from this first wave of data collection have been published elsewhere (Galvin & Richards, 2023). The pre-registration document (osf.io/gdk4x) was updated to reflect the longitudinal component at timepoint 2 (T2) and timepoint 3 (T3). Our main hypothesis was that self-compassion at earlier timepoints would be associated with symptoms of anxiety and depression at later timepoints, but not the reverse, in autistic and non-autistic samples. We also predicted that self-compassion would mediate the association between autistic traits and symptoms of anxiety and depression in both samples.

## Method

This study involved an online survey with participants completing measures three times during a 12-month period (T1 = 0 months, T2 = 6 months, T3 = 12 months). Ethical approval was granted by the first authors’ institution at the start of the study. Participants were asked to provide informed consent at all three timepoints.

### Participants

Autistic and non-autistic adults were recruited using the Prolific recruitment panel (www.prolific.co), and we used the pre-screen feature to select participants based on relevant inclusion/exclusion criteria. Specifically, recruitment involved the use of Prolific pre-screen selection settings for the following participant characteristics: autism diagnosis, no intellectual disability, geography (UK based), and sex (equal male/female recruitment). Autism diagnostic status was therefore confirmed through a combination of Prolific pre-screening information and participant on the day self-report. Two attention check questions were randomly included in the survey to increase the quality of data acquired from the recruitment panel and to ensure participants were reading and engaging with questions appropriately, for example: “This item is to check you are paying attention, please answer with definitely disagree”. Failing both attention checks resulted in the removal of the participant’s data.

In the autistic group, self-report data were available for *n* = 228 individuals at T1, *n* = 156 at T2, and *n* = 165 at T3. In the non-autistic group, *n* = 228 individuals responded at T1, *n* = 122 at T2, and *n* = 124 at T3. The relatively larger sample sizes at T3 compared to T2 could be attributed to T2 data collection taking place during the summer period, whereas T1 data collection took place in January 2022, and T3 data collection took place in January 2023. Although speculative, it is possible that participants had more time and/or were more likely to be online and available to take part in studies during the winter months. All data collected from an individual participant were included in the analyses regardless of whether data were available for all three timepoints. *N* = 278 participants completed both T1 and T2 (*n* = 156 autistic and *n* = 122 non-autistic), and *N* = 228 completed both T2 and T3 (*n* = 136 autistic and *n* = 92 non-autistic). The total number of participants that participated in all three time points was *N* = 226 (*n* = 135 autistic and *n* = 91 non-autistic). To assess the potential impact of attrition, we compared participants within-groups on the main study variables, comparing those who completed all three timepoints with those who did not. No significant differences were found, suggesting that attrition did not have a significant impact on the results.

At baseline (T1), the mean age of autistic participants was 30.5 years (*SD* = 9.47, range = 18–60). The ethnicity of most was White (87.3%), followed by Asian/Asian British (3.9%), Mixed (3.9%), Black/Black British (3.1%), Middle/Near Eastern (1.3%), and Hispanic or Latino (0.4%). One hundred and fifty-seven participants (68.9%) reported a diagnosis of anxiety, 140 (61.4%) depression, 32 (14%) obsessive compulsive disorder (OCD), and 42 (18.4%) attention deficit hyperactivity disorder (ADHD). Some of the remaining autistic participants reported that they suspected having certain conditions but had not received a formal diagnosis: anxiety = 44 (19.3%), depression = 39 (17.1%), OCD = 59 (25.9%), ADHD = 62 (27.2%).

In the non-autistic group, the mean age was 33.1 years (*SD* = 11.07, range = 19–69), and the ethnicity of most was White (85.5%), followed by Asian/Asian British (8.8%), Black/Black British (2.6%), Mixed (1.8%), Black Other (0.4%), Middle/Near Eastern (0.4%), and Hispanic or Latino (0.4%). Sixty-two participants (27.2%) reported a diagnosis of anxiety, 61 (26.8%) depression, 3 (1.3%) OCD, and 4 (1.8%) ADHD. Some remaining non-autistic participants reported no formal diagnosis but suspected they had the following conditions: anxiety = 64 (28.1%), depression = 43 (18.9%), (9.2%), OCD = 31 (13.6%), ADHD = 33 (14.5%).

### Materials

Participants reported their sex (male, female, prefer not to say, prefer to self-describe), age, and whether they were diagnosed with or suspected autism, anxiety, depression, OCD or ADHD. Autistic traits were measured with the Autism Spectrum Quotient (AQ; Baron-Cohen et al., 2001), a 50-item self-report questionnaire. Items are coded 0 (absence of autistic trait) or 1 (presence of autistic trait) and summed to provide an overall score between 0 and 50. The AQ has been shown to be a reliable and valid measure of autistic traits (Woodbury-Smith et al., 2005). Internal consistency (Cronbach’s alpha) in the current study for AQ total score was: T1 α = 0.921, T2 α = 0.926, and T3 α = 0.929.

The Self-Compassion Scale (SCS; Neff, 2003) is a 26-item questionnaire that uses a 5-point scale ranging from 1 (almost never) to 5 (almost always). The SCS produces a total self-compassion score and six subscales. Three subscales are positively scored (self-kindness, common humanity, and mindfulness), and three negatively scored (self-judgement, isolation, and over-identification). In this study we focused on the total self-compassion score only, which is calculated by reverse-scoring the negative subscale items and computing a total mean of all the items. The SCS has been found to be a reliable and valid measure in various clinical samples (e.g., see Costa et al., 2016; Toth-Kiraly & Neff, 2021). Internal consistency for self-compassion total score was: T1 α = 0.942, T2 α = 0.949, and T3 α = 0.951.

The Hospital Anxiety and Depression Scale (HADS; Zigmond & Snaith, 1983) is a 14-item measure to assess anxiety symptoms (7 items) and depression symptoms (7 items) over the previous week. Response options vary across items, but all items are scored on a 0–3 scale. The HADS has been shown to be a reliable and valid measure of anxiety and depression symptoms in autistic people (Uljarevic et al., 2018). Internal consistency values in the current study were as follows: for anxiety, T1 α = 0.867, T2 α = 0.865, and T3 α = 0.863; for depression, T1 α = 0.806, T2 α = 0.830, and T3 α = 0.836.

### Procedure and Data Analysis

The survey was hosted by Qualtrics. To reduce the possibility of missing data, we used the Qualtrics survey function “request response”, which provides reminders to participants about any missed items and highlights these before participants proceed to the next page of the survey. To protect participants’ rights to not respond to any questions that they did not wish to, participants were able to continue with the remainder of the survey without completing said missing items. Given this approach, at the item level, there were minimal missing data (< 1% across all variables for all cases at each timepoint) and this did not differ between groups for any individual variable. In most instances (97% of cases) data were fully complete when a participant had started the survey at a particular timepoint.

We used IBM SPSS version 28 to perform descriptive and correlation analyses. Four cross-lagged models were tested in each group using STATA SE 17.0 (StataCorp, 2021) with Full Information Maximum Likelihood (FIML) estimation to handle missing data. We considered FIML preferable to Multiple Imputation (MI) because research shows that FIML estimates have less bias than MI estimates and that FIML estimates have smaller sampling variance than MI estimates (von Hippel, 2016). With FIML, each parameter is estimated based on all available information from each participant, so even participants with occasional missing data contribute to model estimation. The models evaluated the autoregressive and cross-lagged effects of autistic traits, self-compassion, and symptoms of depression/anxiety across the three waves of data collection. As previous research in non-autistic samples has shown that levels of self-compassion may differ according to age and sex (Neff & Vonk, 2009; Pullmer et al., 2019), these demographics were included as covariates in all models. The following goodness-of-fit indices were used to assess the degree of model fit: comparative fit index (CFI), Tucker-Lewis index (TLI), and root mean square error of approximation (RMSEA). The models were considered to have acceptable fit when CFI > 0.90, TLI > 0.90, and RMSEA < 0.08 (Browne & Cudek, 1993).

## Results

### Preliminary Analyses

Descriptive statistics and comparisons between autistic and non-autistic groups on the main study variables are presented in Table 1. At all three timepoints, autistic participants reported significantly higher autistic traits, anxiety, and depression, and significantly lower self-compassion (all *p*s < 0.001). Partial correlations (controlling for age and sex) are presented in Table 2. At each timepoint, autistic traits and self-compassion were significantly correlated with anxiety and depression at both the cross-sectional and longitudinal level (see Table 2).

**Table 1:** Descriptive statistics and differences between autistic and non-autistic participants for the main study variables

	Autistic			Non-autistic Difference								
	*M*	*SD*				*M*	*SD*		*t*	*df*	*p*	*d*
AQ score T1	33.95	8.38				20.23	7.61		18.30	454	**< 0.001**	**1.71**
AQ score T2	35.69	8.28				21.62	8.59		13.84	276	**< 0.001**	**1.67**
AQ score T3	35.94	8.63				22.35	9.01		13.02	288	**< 0.001**	**1.55**
SCS score T1	2.17	0.64				2.64	0.70		-7.49	454	**< 0.001**	**0.70**
SCS score T2	2.17	0.63				2.71	0.74		-6.54	276	**< 0.001**	**0.79**
SCS score T3	2.17	0.64				2.67	0.77		-6.10	287	**< 0.001**	**0.73**
HADS depression T1	8.72	4.25				6.53	4.01		5.66	454	**< 0.001**	**0.53**
HADS depression T2	8.65	4.63				6.34	3.95		4.40	276	**< 0.001**	**0.53**
HADS depression T3	8.70	4.52				6.51	4.28		4.16	287	**< 0.001**	**0.50**
HADS anxiety T1	12.31	4.73				9.50	4.56		6.43	454	**< 0.001**	**0.60**
HADS anxiety T2	11.85	4.60				9.02	4.60		5.10	276	**< 0.001**	**0.62**
HADS anxiety T3	11.59	4.62				9.43	4.44		4.00	287	**< 0.001**	**0.48**

Bold text indicates a statistically significant difference (*p* < .05, two-tailed test). *AQ* Autism Spectrum Quotient; *SCS* Self Compassion Scale; *HADS* Hospital Anxiety and Depression Scale

**Table 2:** Partial correlations controlling for age and sex. Data for autistic participants are reported below the diagonal and for non-autistic participants above the diagonal

	1	2	3	4	5	6	7	8	9	10	11	12
AQ score T1 (1)		0.839***	0.791***	-0.382***	-0.390***	-0.348***	0.517***	0.431***	0.403***	0.400***	0.358***	0.282**
AQ score T2 (2)	0.799***		0.830***	-0.399***	-0.481***	-0.398***	0.466***	0.453***	0.488***	0.436***	0.425***	0.420***
AQ score T3 (3)	0.840***	0.850***		-0.520***	-0.524***	-0.542***	0.517***	0.481***	0.593***	0.464***	0.517***	0.523***
SCS score T1 (4)	-0.301***	-0.272**	-0.363***		0.761***	0.800***	-0.624***	-0.605***	-0.631***	-0.588***	-0.477***	-0.527***
SCS score T2 (5)	-0.162	-0.304***	-0.249**	0.762***		0.814***	-0.569***	-0.559***	-0.505***	-0.551***	-0.472***	-0.541***
SCS score T3 (6)	-0.202**	-0.187*	-0.304***	0.786***	0.752***		-0.545***	-0.581***	-0.622***	-0.482***	-0.497***	-0.616***
HADS depression T1 (7)	0.168*	0.143	0.148	-0.314***	-0.238**	-0.302***		0.731***	0.690***	0.601***	0.515***	0.439***
HADS depression T2 (8)	0.181*	0.223**	0.165	-0.297***	-0.382***	-0.394***	0.735***		0.739***	0.531***	0.614***	0.501***
HADS depression T3 (9)	0.195*	0.255**	0.211*	-0.333***	-0.372***	-0.445***	0.640***	0.789***		0.541***	0.626***	0.677***
HADS anxiety T1 (10)	0.285***	0.229**	0.310***	-0.410***	-0.261**	-0.402***	0.593***	0.491***	0.432***		0.726***	0.681***
HADS anxiety T2 (11)	0.227**	0.170*	0.195*	-0.353***	-0.379***	-0.384***	0.486***	0.591***	0.586***	0.720***		0.759***
HADS anxiety T3 (12)	0.191*	0.229**	0.296***	-0.421***	-0.422***	-0.502***	0.511***	0.589***	0.661***	0.706***	0.803***	

*Note. AQ* Autism Spectrum Quotient, *SCS* Self Compassion Scale, *HADS* Hospital Anxiety and Depression Scale

*** *p* < .001, **p < .01, *p < .05

### Cross-lagged Model of Depression in Autistic Adults

**Fig. 1:**
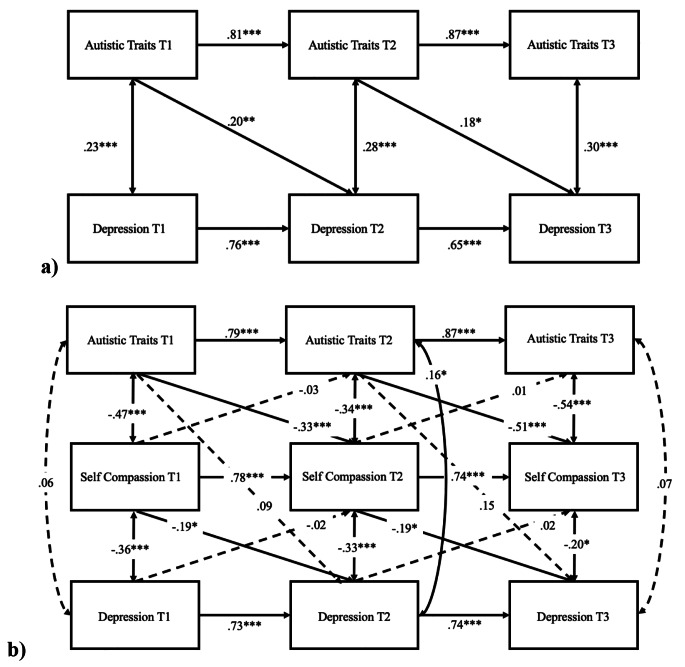
Cross-lagged models (autistic sample) of (**a**) autistic traits and depression, and (**b**) including self-compassion to evaluate the direct and indirect associations. *Note*: sex and age were controlled. Standardised coefficients are presented. Dashed line represents non-significant path. ***p < .001, **p < .01, *p < .05

In autistic adults, the cross-lagged model of autistic traits and depression, controlling for age and sex (Fig. 1a), showed adequate model fit, CFI = 0.98, TLI = 0.97, RMSEA = 0.02. The autoregressive effects were significant for both autistic traits and depression (all *p*s < 0.001). T1 autistic traits significantly predicted T2 depression (*β* = 0.20, 95% CIs [0.07, 0.33], *p* = .001), and T2 autistic traits significantly predicted T3 depression (*β* = 0.18, 95% CIs [0.02, 0.33], *p* = .03). The cross-lagged model of autistic traits, self-compassion, and depression (Fig. 1b) showed satisfactory model fit, CFI = 0.97, TLI = 0.96, RMSEA = 0.03. The autoregressive effects were significant for autistic traits, self-compassion, and depression (all *p*s < 0.001). Autistic traits predicted subsequent self-compassion from T1 to T2 (*β* = − 0.33, 95% CIs [-0.17, − 0.48], *p* < .001) and from T2 to T3 (*β* = − 0.51, 95% CIs [-0.32, − 0.70], *p* < .001). Earlier self-compassion predicted subsequent depression from T1 to T2 (*β* = − 0.19, 95% CIs [-0.03, − 0.35], *p* = .02) and from T2 to T3 (*β* = − 0.19, 95% CIs [-0.03, − 0.36], *p* = .02), but earlier depression did not predict subsequent self-compassion from T1 to T2 (*β* = − 0.02, 95% CIs [-0.12, 0.08], *p* = .68) or T2 to T3 (*β* = 0.02, 95% CIs [-0.10, 0.12], *p* = .70). Furthermore, the indirect role of T2 self-compassion within the association between T1 autistic traits and T3 depression was confirmed: a significant mediation effect was found, such that T2 self-compassion mediated the effect of T1 autistic traits on T3 depression (*β* = 0.09, 95% CIs [0.04, 0.11], *p* = .001).

### Cross-lagged Model of Anxiety in Autistic Adults

**Fig. 2:**
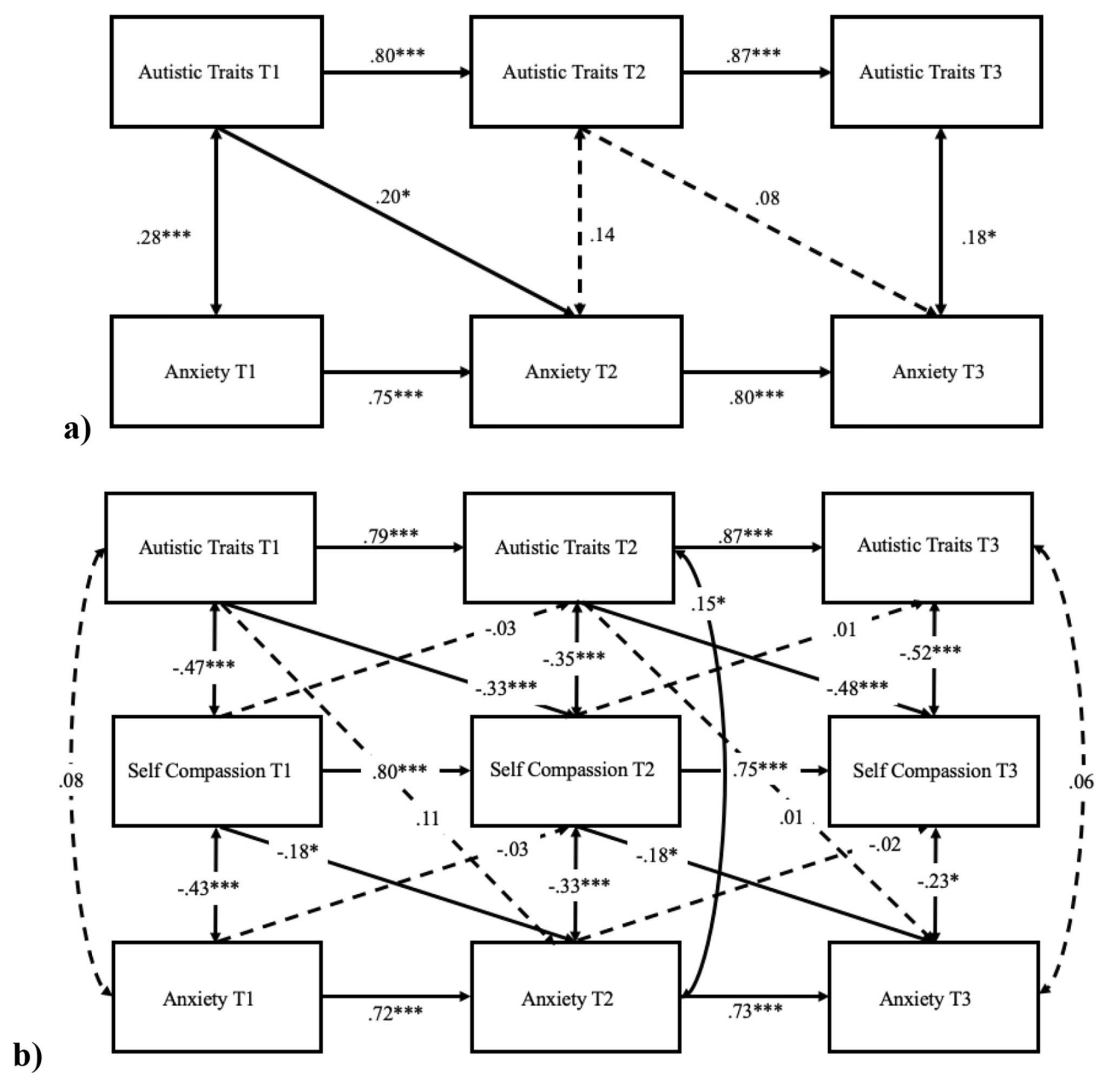
Cross-lagged models (autistic sample) of (**a**) autistic traits and anxiety, and (**b**) including self-compassion to evaluate the direct and indirect associations. *Note*: sex and age were controlled. Standardised coefficients are presented. Dashed line represents non-significant path. ***p < .001, **p < .01, *p < .05

The cross-lagged model of autistic traits and anxiety, controlling for age and sex (Fig. 2a), showed adequate model fit, CFI = 0.99, TLI = 0.97, RMSEA = 0.02. The autoregressive effects were significant for both autistic traits and anxiety (all *p*s < 0.001). T1 autistic traits significantly predicted T2 anxiety (*β* = 0.20, 95% CIs [0.03, 0.37], *p* = .02). However, no such relationship was found between T2 autistic traits and T3 anxiety (*β* = 0.08, 95% CIs [-0.11, 0.27], *p* = .43). The cross-lagged model of autistic traits, self-compassion, and anxiety (Fig. 2b) showed satisfactory model fit, CFI = 0.97, TLI = 0.91, RMSEA = 0.05. The autoregressive effects were significant for autistic traits, self-compassion, and anxiety (all *p*s < 0.001). Autistic traits predicted subsequent self-compassion from T1 to T2 (*β* = − 0.33, 95% CIs [-0.17, − 0.48], *p* < .001) and from T2 to T3 (*β* = − 0.48, 95% CIs [-0.28, − 0.67], *p* < .001). Earlier self-compassion predicted subsequent anxiety from T1 to T2 (*β* = − 0.18, 95% CIs [-0.02, − 0.36], *p* = .04) and from T2 to T3 (*β* = − 0.18, 95% CIs [-0.03, − 0.36], *p* = .02), but earlier anxiety did not predict subsequent self-compassion from T1 to T2 (*β* = − 0.03, 95% CIs [-0.07, 0.13], *p* = .53) or T2 to T3 (*β* = − 0.02, 95% CIs [-0.09, 0.12], *p* = .66). Furthermore, the indirect role of T2 self-compassion within the association between T1 autistic traits and T3 anxiety was statistically significant (*β* = 0.10, 95% CIs [0.07, 0.13], *p* < .001).

### Cross-lagged Model of Depression in Non-autistic Adults

**Fig. 3:**
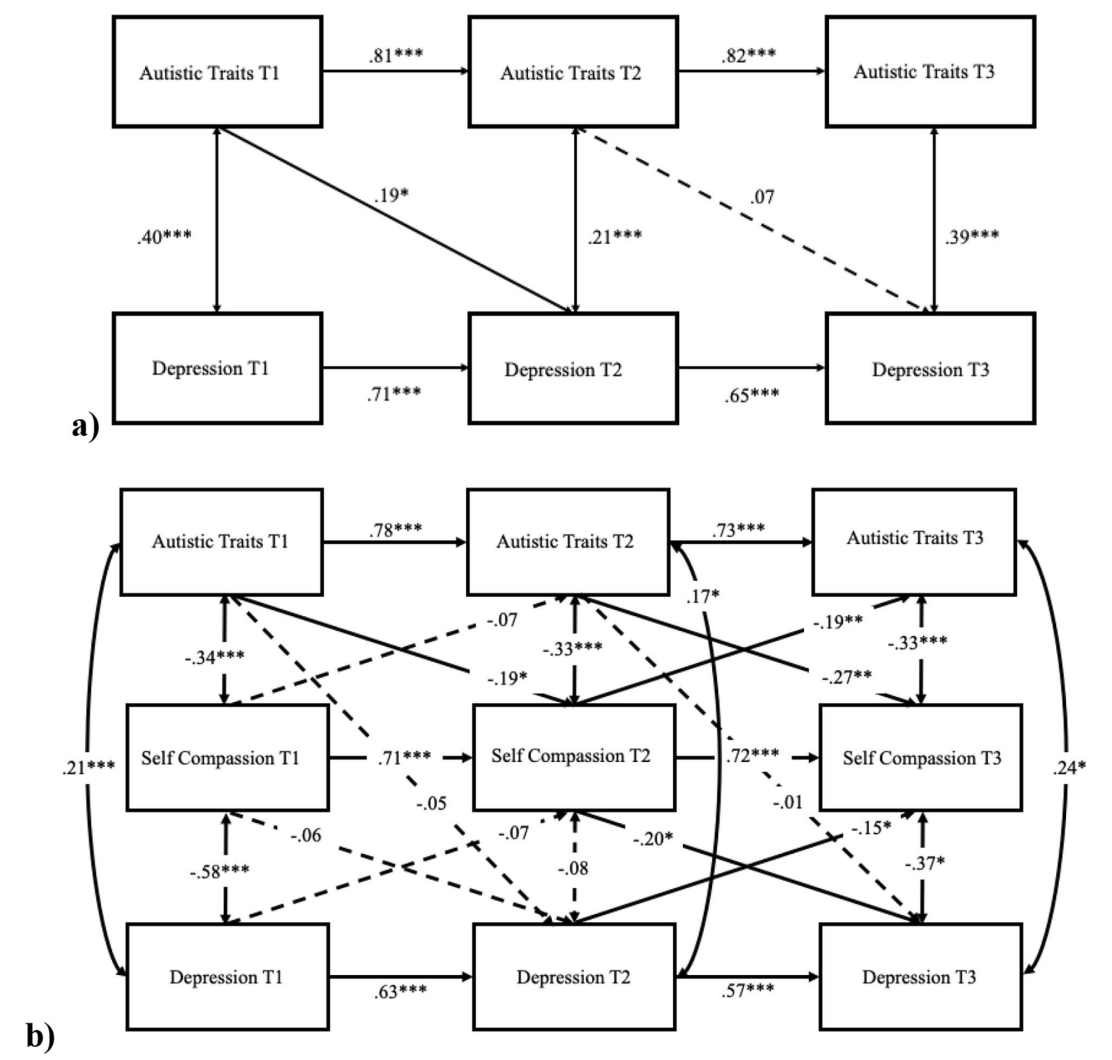
Cross-lagged models (non-autistic sample) of (**a**) autistic traits and depression, and (**b**) including self-compassion to evaluate the direct and indirect associations. *Note*: sex and age were controlled. Standardised coefficients are presented. Dashed line represents non-significant path. ***p < .001, **p < .01, *p < .05

In the non-autistic sample, the cross-lagged model of autistic traits and depression, controlling for age and sex (Fig. 3a), showed adequate model fit, CFI = 0.98, TLI = 0.97, RMSEA = 0.02. The autoregressive effects were all statistically significant (all *p*s < 0.001). T1 autistic traits significantly predicted T2 depression (*β* = 0.19, 95% CIs [0.02, 0.40], *p* = .03), but T2 autistic traits did not significantly predict T3 depression (*β* = 0.07, 95% CIs [-0.25, 0.12], *p* = .48). The cross-lagged model of autistic traits, self-compassion, and depression (Fig. 3b) showed overall satisfactory model fit, although TLI fell just short of the > 0.90 threshold: CFI = 0.95, TLI = 0.89, RMSEA = 0.06. The autoregressive effects were significant for all variables at all timepoints (all *p*s < 0.001). Autistic traits predicted subsequent self-compassion from T1 to T2 (*β* = − 0.19, 95% CIs [-0.03, − 0.36], *p* = .02) and from T2 to T3 (*β* = − 0.27, 95% CIs [-0.09, − 0.44], *p* = .002). Earlier self-compassion did not predict future depression from T1 to T2 (*β* = − 0.06, 95% CIs [-0.25, 0.14], *p* = .57), but a significant effect was found from T2 to T3 (*β* = − 0.20, 95% CIs [-0.02, − 0.40], *p* = .04). Earlier depression did not predict future self-compassion from T1 to T2 (*β* = − 0.07, 95% CIs [-0.17, 0.04], *p* = .21), but it did from T2 to T3 (*β* = − 0.15, 95% CIs [-0.03, − 0.26], *p* = .02). A significant mediation effect was found, such that T2 self-compassion mediated the effect of T1 autistic traits on T3 depression (*β* = 0.07, 95% CIs [0.03, 0.13], *p* = .009).

### Cross-lagged Model of Anxiety in Non-autistic Adults

**Fig. 4:**
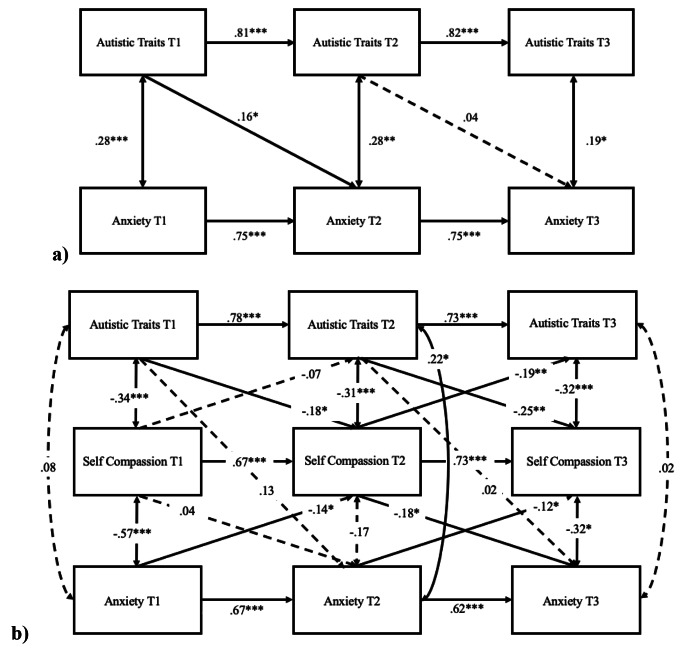
Cross-lagged models (non-autistic sample) of (**a**) autistic traits and anxiety and (**b**) including self-compassion to evaluate the direct and indirect associations. *Note*: sex and age were controlled. Standardised coefficients are presented. Dashed line represents non-significant path. ***p < .001, **p < .01, *p < .05

Finally, the cross-lagged model of autistic traits and anxiety in non-autistic adults, controlling for age and sex (Fig. 4a), showed adequate model fit, CFI = 0.97, TLI = 0.96, RMSEA = 0.03. The autoregressive effects were all significant (all *p*s < 0.001). T1 autistic traits significantly predicted T2 anxiety (*β* = 0.16, 95% CIs [0.02, 0.33], *p* = .04), but T2 autistic traits did not significantly predict T3 anxiety (*β* = 0.04, 95% CIs [-0.24, 0.17], *p* = .74). The cross-lagged model of autistic traits, self-compassion, and anxiety (Fig. 4b) showed adequate model fit: CFI = 0.96, TLI = 0.90, RMSEA = 0.05. The autoregressive effects were all statistically significant (all *p*s < 0.001). Autistic traits predicted future self-compassion from T1 to T2 (*β* = − 0.18, 95% CIs [-0.03, − 0.34], *p* = .02) and from T2 to T3 (*β* = − 0.25, 95% CIs [-0.08, − 0.42], *p* = .002). Earlier self-compassion did not predict future anxiety from T1 to T2 (*β* = − 0.04, 95% CIs [-0.15, 0.23], *p* = .70), but a significant effect was found from T2 to T3 (*β* = − 0.18, 95% CIs [-0.01, − 0.36], *p* = .04). Interestingly, earlier anxiety significantly predicted future self-compassion from T1 to T2 (*β* = − 0.14, 95% CIs [-0.02, − 0.27], *p* = .02) and T2 to T3 (*β* = − 0.12, 95% CIs [-0.00, − 0.23], *p* = .04). Finally, a significant mediation effect was found, such that T2 self-compassion mediated the effect of T1 autistic traits on T3 anxiety (*β* = 0.10, 95% CIs [0.04, 0.18], *p* = .003).

## Discussion

The current study explored the longitudinal associations between autistic traits, self-compassion, anxiety, and depression in a sample of autistic and non-autistic adults from the UK. In the autistic sample, a consistent pattern emerged: low levels of self-compassion consistently predicted subsequent anxiety and depression across all models and at all assessment points. Conversely, the reverse relationship, where earlier anxiety and depression predicted later self-compassion, did not exhibit a corresponding trend. This finding supports our original hypothesis and corroborates previous research conducted in the general population (Pullmer et al., 2019; Stutts et al., 2018). Importantly, this is the most convincing evidence to date that earlier self-compassion might be important for future anxiety and depression in autistic people.

In the non-autistic sample, mixed results were observed, with low self-compassion predicting subsequent anxiety and depression between T2 and T3 but not between T1 and T2. Furthermore, T1 anxiety predicted T2 self-compassion, and T2 anxiety and T2 depression predicted T3 self-compassion. While these findings suggest bi-directional relationships within the non-autistic sample, it is important to note that, across all timepoints, earlier self-compassion more strongly predicted future anxiety/depression symptoms than vice versa. These findings thus partially support previous research, and, importantly, maintain the view that low self-compassion serves as a strong predictor of anxiety and depression symptoms in general populations. To some extent, the bi-directional findings in the non-autistic sample echo findings reported by You et al. (2017), who observed that earlier non-suicidal self-injury predicted subsequent self-criticism in adolescents in a three-wave study with a 6-month lag. Additionally, Pastore et al. (2022) found a bi-directional relationship between self-compassion and happiness in a longitudinal general population study, while Veilleux et al. (2023) identified a bi-directional relationship between self-criticism and negative affect.

Of particular interest is that the data in this study showed a consistent mediating pattern, in that autistic traits (T1) predicted later self-compassion (T2), which further predicted later anxiety (T3) and depression (T3). This finding is conceptually consistent with prior cross-sectional research (Galvin et al., 2021; Galvin & Richards, 2023), and provides additional support to the idea that self-compassion acts as a mediating variable between autistic traits and anxiety/depression. Moreover, once self-compassion entered the model, the direct effects of autistic traits on future anxiety and depression lost statistical significance in all models, for both autistic and non-autistic samples. In essence, these findings suggest that autistic traits indirectly influence subsequent depression and anxiety through reduced self-compassion in both autistic and non-autistic adults. A clinically relevant interpretation of these findings is that the severity of autistic traits may exacerbate or lead to mental health problems via low self-compassion. This knowledge can inform the development of interventions that specifically target self-compassion as a means to enhance the mental well-being of both autistic and non-autistic adults with high levels of autistic traits.

The current study’s findings align with prior cross-sectional research that shows a negative relationship between autistic traits and self-compassion (Cai et al., 2023a; Galvin & Richards, 2023). However, it is important to note that the biopsychosocial factors which may explain this relationship remain unknown. According to a compassion-based theory of autism and mental health proposed by Galvin (2023), i.e., the “Compassionate Brain Theory”, reduced self-compassion in individuals with high levels of autistic traits or diagnosed autism may originate in early childhood and be intricately linked to attachment processes and increased adverse experiences across the lifespan. In autistic individuals, such experiences may include heightened exposure to trauma (Dodds, 2021), instances of bullying (Zablotsky et al., 2014), the necessity to camouflage one’s autistic traits (Cook et al., 2021), and a pervasive sense of non-acceptance from others (Cage et al., 2018).

In the current study, it was particularly interesting that there were no instances where anxiety/depression predicted future self-compassion in autistic adults. If this is confirmed in future studies, one idea is the possibility of a missing or reduced feedback loop. Without the self-regulating action of a negative feedback loop between self-compassion and psychopathology in autistic or high trait individuals, low self-compassionate responses may spiral out of control and result in further escalation of symptoms. This possibility could be a useful line of inquiry for future research.

In addition to the above, the current findings contribute to the growing body of evidence suggesting that autistic traits are associated with subsequent mental health symptoms (Hou & Shi, 2023; Rai et al., 2018). Within the autistic sample, higher levels of autistic traits at earlier timepoints predicted increased depression between T1 and T2 and between T2 and T3. Additionally, they predicted heightened anxiety between T1 and T2, although this effect was not present between T2 and T3. Within the non-autistic sample, earlier autistic traits predicted increased future anxiety and depression between T1 and T2, but not between T2 and T3. It is difficult to determine why autistic traits did not predict anxiety after T2 in the autistic sample, or anxiety or depression after T2 in the non-autistic sample, but we suspect it could be due to sample size, measurement timing, or other mediating factors potentially influencing this relationship.

Future research should examine self-compassion alongside other previously established mediating factors, such as alexithymia and emotion regulation (Morie et al., 2019; Vuillier et al., 2020), to better understand the role of self-compassion in the mental health experiences of autistic people. Cai et al. (2023b) found cross-sectional support for an indirect effect in the association between low self-compassion and anxiety/depression via emotion dysregulation, but no support for an indirect path between emotion dysregulation and anxiety/depression via low self-compassion. This is important because, if these findings are confirmed longitudinally, it would suggest that low levels of self-compassion may not only precede future mental health symptoms, but also other difficulties associated with autism. Therefore, cultivating self-compassion in autistic people could positively influence a range of psychological processes and outcomes.

The findings of the current study should be interpreted with a few limitations in mind. First, since the study relied on an online survey it was not possible to independently verify the participants’ autism diagnostic status using more established tools like the Autism Diagnostic Observation Schedule (Lord et al., 2000) or Autism Diagnostic Interview-Revised (Rutter et al., 2003). Nonetheless, we used a combination of the Prolific pre-screen information and on the day self-report to determine diagnostic status, thus providing more confidence than on the day self-report alone. Furthermore, the AQ has good sensitivity and specificity (Ruzich et al., 2015), and the scores obtained in the current study were consistently higher in the autistic group at each timepoint, with very large effect sizes (Cohen’s *d* > 1.5). A second limitation is reliance on self-report questionnaires, which introduce potential for response bias that could compromise validity of the data (Adams et al., 1999). Relatedly, attention check questions were created by the authors for the purpose of this study, but these have not been previously validated.

While the current study was also constrained by a limited sample size, further investigations with larger samples could provide more insights into the within-person mechanisms underlying the observed associations by utilising random intercept cross-lagged panel models. A further issue is that exclusion of individuals with an intellectual disability limits the generalisability of the findings to the full spectrum of intellectual functioning. In addition, while the participant characteristics were controlled for the purpose of this study, this resulted in a lack of diversity in the sample. Future research could look at self-compassion and intersectionality. Finally, another possible issue is that we have no information on participants’ knowledge and understanding of self-compassion. Considering there was a high prevalence of respondents with co-occurring mental health conditions, particularly in the autistic sample, it would not be surprising if many have experience of psychological therapy and might therefore have used and/or currently be using self-compassion techniques. Future research could control for this in the study design.

Notwithstanding the above limitations, the findings of the current study make significant theoretical and practical contributions. Specifically, the study extends mental health research in the context of autism by confirming the beneficial effects of self-compassion on depression and anxiety, and by supporting findings from previous studies in general populations (Ferrari et al., 2019; Macbeth & Gumley, 2012; Wilson et al., 2019). Furthermore, we have provided additional support for the mediating role of self-compassion in connecting autistic traits with anxiety and depression, thus enhancing researchers’ and clinicians’ understanding of why autistic traits may influence these mental health outcomes. Overall, considering the high prevalence of anxiety and depression among autistic people (Hollocks et al., 2019; Lugo-Marin et al., 2019), and given the fact that self-compassion can be cultivated through practice (Gilbert, 2009; Neff, 2003), the current findings suggest that self-compassion could be a useful therapeutic target to promote mental health in the autistic population.

## Data Availability

Available on the Open Science Framework.
